# An extinction event in planktonic Foraminifera preceded by stabilizing selection

**DOI:** 10.1371/journal.pone.0223490

**Published:** 2019-10-14

**Authors:** Manuel F. G. Weinkauf, Fabian G. W. Bonitz, Rossana Martini, Michal Kučera

**Affiliations:** 1 Department of Geosciences, Eberhard–Karls Universität Tübingen, Tübingen, Germany; 2 Center for Marine Environmental Sciences (MARUM), Universität Bremen, Bremen, Germany; 3 Department of Earth Sciences, Université de Genève, Genève, Switzerland; MARE - Marine and Environmental Sciences Centre, PORTUGAL

## Abstract

Unless they adapt, populations facing persistent stress are threatened by extinction. Theoretically, populations facing stress can react by either disruption (increasing trait variation and potentially generating new traits) or stabilization (decreasing trait variation). In the short term, stabilization is more economical, because it quickly transfers a large part of the population closer to a new ecological optimum. However, stabilization is deleterious in the face of persistently increasing stress, because it reduces variability and thus decreases the ability to react to further changes. Understanding how natural populations react to intensifying stress reaching terminal levels is key to assessing their resilience to environmental change such as that caused by global warming. Because extinctions are hard to predict, observational data on the adaptation of populations facing extinction are rare. Here, we make use of the glacial salinity rise in the Red Sea as a natural experiment allowing us to analyse the reaction of planktonic Foraminifera to stress escalation in the geological past. We analyse morphological trait state and variation in two species across a salinity rise leading to their local extinction. *Trilobatus sacculifer* reacted by stabilization in shape and size, detectable several thousand years prior to extinction. *Orbulina universa* reacted by trait divergence, but each of the two divergent populations remained stable or reacted by further stabilization. These observations indicate that the default reaction of the studied Foraminifera is stabilization, and that stress escalation did not lead to the emergence of adapted forms. An inherent inability to breach the global adaptive threshold would explain why communities of Foraminifera and other marine protists reacted to Quaternary climate change by tracking their zonally shifting environments. It also means that populations of marine plankton species adapted to response by migration will be at risk of extinction when exposed to stress outside of the adaptive range.

## Introduction

Imminent extinctions, that result from persistent environmental perturbations which are unbeneficial for an incumbent population, are difficult to study due to their unpredictability in recent environments [1]. This is because it is difficult to quantify stress reactions over ecologically relevant time-scales, which cannot be simulated in the laboratory, or because the severity and ultimate outcome of the stress reaction is hard to predict.

Mechanistically, environmental stress influences population morphology by decreasing the fitness of specimens which show a high degree of developmental instability [2,3]. On the population level, this can lead to either stabilizing selection when a certain phenotype is the preferred survivor toward the stress [4] or disruptive selection when higher variation better guarantees the survival of the population in a rapidly changing environment [5]. Both stabilizing and disruptive selection are detectable by assessing the phenotypic variation (i.e. the range of realized phenotypes [6]) of the population, which can therefore be used as a measure for developmental stability. Shape and size of organisms have long been hypothesized to reflect the influence of environmental stress on the physiology of an organism during its lifetime [2,7–10]. Therefore, a characterization of shape and size and their variation should in principle allow an assessment of the severity of stress exposure. Under this assumption, stress exposure that leads to extinction, can be expected to leave a discernible imprint on morphology in pre-extinction populations [11].

In this regard, the sedimentary record offers a unique opportunity to study the effects of terminal stress levels (i.e. stress leading to local extinction), because here the outcome can be directly observed. This benefit comes at the cost that the sedimentary record always comprises a temporally integrated sequence, and that the environmental change that caused the local extinction can be difficult to reconstruct in some settings [12]. Shell bearing protists, such as planktonic Foraminifera, are perfect model systems for such studies, because they are preserved in great numbers in the fossil record [13,14] and thus allow a robust analysis of their variation on the population level.

Here, we use two species of planktonic Foraminifera from the Red Sea sediment core KL09 (*c*.450 kyrs BP) to study their morphological reaction toward terminal stress levels. Understanding the morphological reaction of Foraminifera toward environmental stress could serve as a proxy for evolvability of the present assemblages in this organismal group. These observations can potentially be even applied to the microplankton at large, assuming evidence that evolutionary patterns in the protist realm differ from those of higher organisms but seem to be bound to their own laws across all plankton [15–17]. Past studies have shown that morphological deviations in benthic and planktonic Foraminifera can be caused by environmental forcing [12,18–24]. But since these studies quantified morphology in very different ways, the results obtained are scarce and controversial. In light of earlier results, it is reasonable to assume to see a morphological trend in the assemblage of planktonic Foraminifera associated with terminal stress levels. We hypothesize that a correlation between morphology and either environmental proxies or species abundance (as biotic indicator for the stress level the community is exposed to) exists. To test this hypothesis, we use Pleistocene sediments from the Red Sea, where several species of planktonic Foraminifera regionally disappeared from the fossil record (hereafter called local extinction) within aplanktonic zones resulting from environmental change [25]. Shortly before the onset of each aplanktonic zone, a distinct sequential local extinction pattern of different planktonic Foraminifera species can be observed, until virtually all planktonic Foraminifera are absent from the fossil record. In contrast to comparable cases where fossil material is used [12,22], we are here in the unique situation that the extinction events can nearly exclusively be linked to salinity increase [26,27], allowing a qualification of the stressor which acted on the assemblage.

The study will evaluate the value of morphology as proxy for stress and the adaptive patterns acting in planktonic Foraminifera communities. By qualifying evolutionary patterns in regard to environmental stress reactions, we will be able to gain insights into the evolutionary mode acting upon planktonic Foraminifera, and to evaluate their adaptive potential to environmental change.

## Material and methods

### Sample material

**Fig 1:**
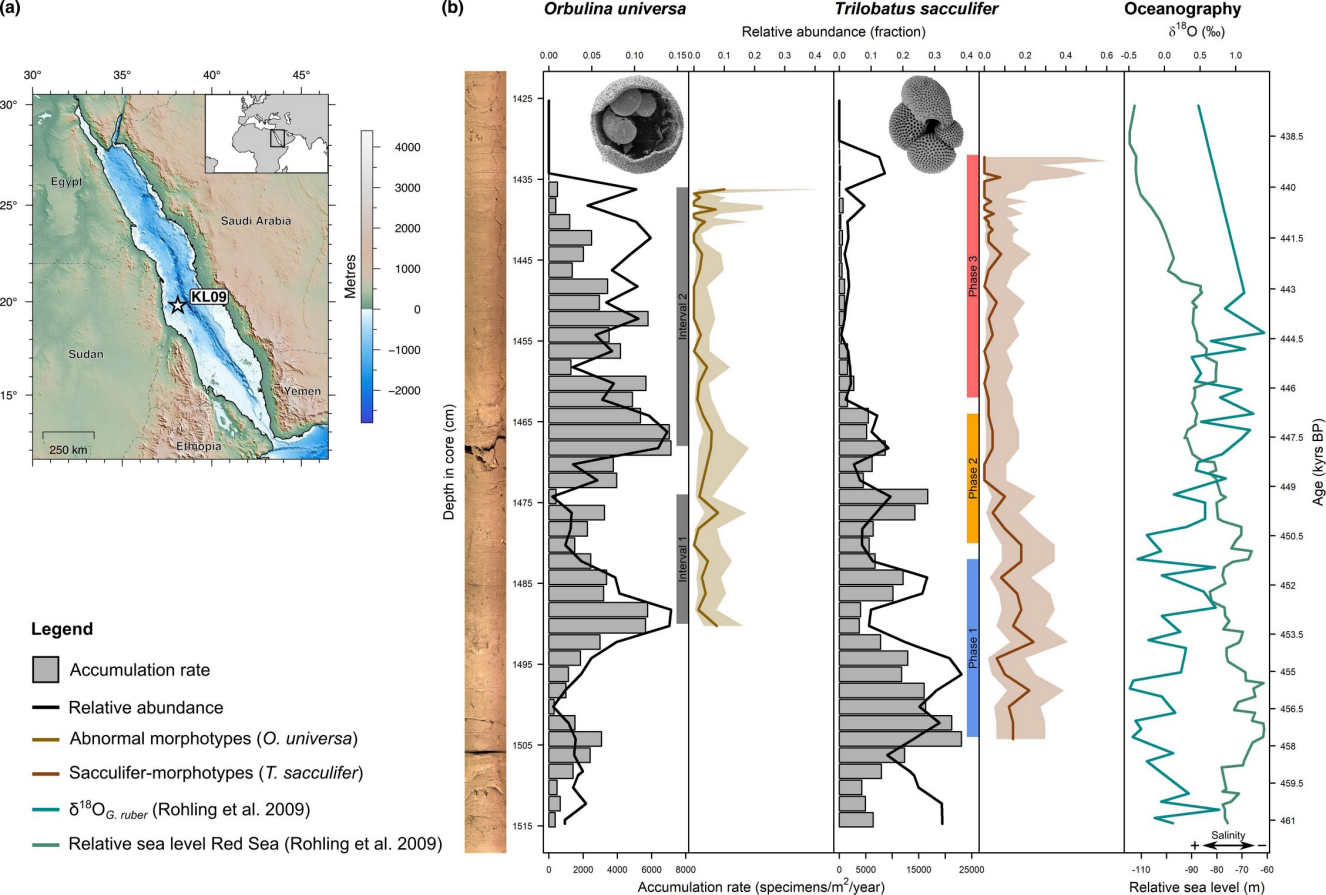
Summary of the sampling material from piston core Geo-TÜ KL09. (a) Map of the sampling area with indication of the core position. (b) Stratigraphic plot with core image. Accumulation rates, relative abundances in relation to other species of planktonic Foraminifera, and the incidence of abnormal morphotypes in *Orbulina universa* and sacculifer-morphotypes in *Trilobatus sacculifer* is indicated (shaded area depicts 95% confidence intervals). The aplanktonic zone begins at approximately 438 kyrs BP. The two intervals of dropping abundance for *O*. *universa* (Intervals 1 and 2) and the three phases defined for *T*. *sacculifer* (Phases 1–3) based on relative abundances are indicated. The δ^18^O_G. ruber_ measurements and the three-point moving average relative sea level in the Red Sea [26] is also shown. Example scanning electron microscope images of the two investigated species are shown in the respective panels. The *O*. *universa* specimen was cracked open to reveal the juvenile shell inside. Image of *T*. *sacculifer* from Hesemann [33].

For the present study we used material from piston core Geo-TÜ KL09 (19.804° N, 38.103° E; Fig 1) taken in the Red Sea during the RV Meteor cruise M5-2 [28]. We chose material from marine isotope stage 12 (MIS 12), specifically the range from 461.1 to 437.5 kyrs BP (age model after Grant, Rohling [29]), to investigate planktonic Foraminifera morphology under terminal environmental stress. The interval covers an aplanktonic zone, which occurred during the Pleistocene in the Red Sea as a result of extremely high salinities (>49 on the psu scale) induced by a changed circulation pattern in the Red Sea basin [25]. The MIS 12 aplanktonic zone has been chosen, because it is the most prominent and longest one preserved in KL09. The spatial resolution for our sampling varies between 0.5 and 2 cm, for a temporal resolution of 120–500 years (higher resolution for the last 2000 years before the aplanktonic zone). Since salinity values in the Red Sea are tightly coupled with the relative sea level and δ^18^O values of the sea water [27], and high resolution sea level and stable isotope reconstructions from the Red Sea exist [26] (Fig 1), we can approximate past sea water salinity to test for its influence on foraminiferal shell morphology. While MIS 12 represents a glacial period, it was shown that the sea surface temperature in the Red Sea area rarely dropped below 24°C [30], which is well within the temperature tolerance levels of all species common in the Red Sea [31,32], so that sea surface temperature played no considerable role in environmental stress levels.

We investigated two abundant, symbiont-bearing species of planktonic Foraminifera (Fig 1, S1 File). Both species react sensitively to salinity changes, as shown by their consistently early position within the extinction sequence of the aplanktonic zones [25]. *Orbulina universa* d’Orbigny, 1839 is characterized by a trochospiral juvenile shell that is overgrown by a spherical terminal chamber. *Trilobatus sacculifer* (Brady, 1877) Spezzaferri et al., 2015 shows a trochospiral shell, in which the terminal chamber sometimes develops a sac-like shape. Both species are surface dwellers, partly due to their symbionts’ need for light, and thus occur in comparable environments [34–36]. Differences in their reactions can therefore not be the result of the exposure to considerably different environmental forcing.

### Sample preparation and data acquisition

Sediment samples of 0.5 cm thickness were taken with a U-channel from piston core Geo-TÜ KL09, dried in an oven at 50°C, soaked in tap-water, and washed over a 63 μm screen under flowing tap-water. The residual >63 μm was dried and dry-sieved over a 150 μm screen. Only the fraction >150 μm was used for this study to ensure that the analysed individuals would have reached their adult stage so that an ontogenetic effect on the shape analyses could be widely eliminated [37]. For census counts, only a small fraction of each sample (split with a microsplitter) has been used. Aliquots containing at least 300 specimens were investigated for their species composition and counted samples were stored without modification.

Planktonic foraminiferal specimens from representative aliquots (split with a microsplitter) were picked with a needle and transferred onto glass slides, were they were fixed in position using permanent glue. We were striving for a sample size *n* of at least 50 specimens per sample. For *O*. *universa* we always used the entire sample aliquot, while for *T*. *sacculifer* a randomly selected subsample (*n* = 50) was analysed due to the more time-consuming analysis in this species. Images for morphological analyses were taken with a Canon EOS 500D digital mirror reflex camera attached to a Zeiss Stereo.V8 binocular microscope under constant magnification.

Morphological data for *O*. *universa* (2774 specimens) were semi-automatically extracted from high-contrast transmitted light images using the software FIJI (ImageJ v. 1.48s, [38]). We used the shell size (Feret diameter) and shell roundness (ratio between longest and shortest axis of a fitted ellipse, 1.0 equals a sphere) (S1 File). We also counted the incidence of the abnormal morphotypes ‘*Orbulina suturalis*’ and ‘*Biorbulina bilobata*’, which have been shown in laboratory experiments to be ecophenotypes of the same biological species [39,40]. For *T*. *sacculifer* (2228 specimens), images were taken under reflected light with specimens oriented such that the apertural plane was lying horizontally, perpendicular to the direction of view (apertural standard view). In these images, 12 landmarks (S1 File) were manually digitized in R v. 3.5.1 [41]. In 68 specimens, parts of the structures were not visible clearly enough to extract all landmarks and they were excluded from all analyses using the landmark data, leaving a dataset of 2160 specimens. Furthermore, the attribution to one of the three morphotypes of the species (trilobus, quadrilobatus, sacculifer; compare André, Weiner [42]) was recorded. Attribution to a morphotype was done by the same author (MFGW) for all specimens to avoid errors due to varying species concepts.

### Morphometric data analysis

All statistical analyses have been performed in R v. 3.5.1 [41]. The normality of data distribution was tested with a Shapiro–Wilk test [43] and the homoscedasticity by a Fligner–Killeen test [44] wherever necessary. Confidence intervals of morphological parameters were calculated via bootstrapping with the R-package ‘boot’ v. 1.3–20 [45]. Confidence intervals for morphotype occurrences were calculated using multinomial equations [46]. In all cases of multiple testing (e.g. pairwise tests between more than two groups), *p*-values were corrected for the false discovery rate after Benjamini and Yekutieli [47].

To investigate trends in the incidence of abnormal morphotypes of *O*. *universa* and sacculifer-morphotypes of *T*. *sacculifer* over time, we used generalized linear models (GLM, [48]) on the binomial distribution with logit as link-function. The coefficient of determination was determined according to equations by Nagelkerke [49] (Nagelkerke-*R*^2^). For *T*. *sacculifer* we additionally used pairwise two-proportions *z*-tests to investigate the influence of stress levels on the incidence of sacculifer-morphotypes.

For *O*. *universa*, the extracted morphological parameters were subjected to traditional morphometric analyses. For *T*. *sacculifer* we used traditional morphometrics as well as geometric morphometric analytical methods as described in Claude [50] and Zelditch, Swiderski [51]. The landmark coordinates were fully Procrustes fitted using the R-package ‘shapes’ v. 1.2.4. The centroid sizes were used as size parameters for *T*. *sacculifer* shells. All size data were log_*e*_-transformed prior to analyses.

Traditional morphometric group-differences were investigated by a Kruskal–Wallis test [52], under circumstances followed by pairwise Mann–Whitney *U* tests [53]. Bimodality was tested using Hartigan’s dip test [54] as implemented in the R-package ‘diptest’ v. 0.75–7, and the bimodality coefficient after Ellison [55] was calculated with the R-package ‘modes’ v. 0.7.0. Trends in variation were analysed by calculating the coefficient of variation with the 95% confidence interval after Vangel [56]. Variation trends were additionally tested using a randomization approach, where the observed trend of morphological variation was compared to a random resampling of specimens (with replacement, 1000 replications) with sample sizes kept as in the original data. Kendall–Theil robust line fitting model III linear regression [57–59] was used for all linear regression analyses. To test for correlations between size and roundness in *O*. *universa* we applied a Kendall rank-order correlation [57].

The shape of *T*. *sacculifer* specimens, defined as the Riemannian shape distance [60] of an individual landmark configuration from the grand mean shape, was investigated using geometric morphometrics. The variation of the *T*. *sacculifer* populations was approximated as variance of individual Riemannian shape distances within the population. Its confidence intervals and standard errors were derived by bootstrapping, and pairwise comparisons were performed by Student’s *t*-tests with adjusted degrees of freedom [number of specimens in both groups minus two; 51]. Superimposed landmark data were analysed for differences between groups using non-parametric multivariate analysis of variance (NPMANOVA; [61]) on the Euclidean distances with 999 permutation as implemented in the R-package ‘vegan’ v. 2.5–2. Ensuing pairwise comparisons used the ‘testmeanshapes’ function of the R-package ‘shapes’ v. 1.2.4 with 999 permutations. Shape changes were visualized using thin-plate splines [62]; and canonical variates analysis (CVA, [63]) from the R-package ‘MASS’ v. 7.3–50 [64] was used to analyse the shape changes between predefined groups.

We further tested all observed morphological trends against three potential models of phyletic evolution using the R-package ‘paleoTS’ v. 0.5–1 [65]. This allows to distinguish between directional selection (general random walk), a directional pattern due to the accumulation of random change (unbiased random walk), and a system that does not change over time (stasis). The corrected Akaike information criterion (AICc, [66]) in combination with Akaike weights [67] was used to decide, which model best describes the data.

## Results

### Abundance patterns

#### Species abundances

*Orbulina universa* occurred at generally low abundances (5.6% on average) that never exceeded 14.5% of the total assemblage (Fig 1). The species shows two abundance peaks in the studied interval, in case of the second event leading to local extinction. The first abundance peak occurred around 453 kyrs BP, and was followed by a rapid decline in abundance until 449.5 kyrs BP. Abundances then rose again until 447.5 kyrs BP, after which a second decline was observed, which culminated in the local extinction of the species between 440.0 and 439.1 kyrs BP. Based on the abundance, we could separate the *O*. *universa* population into two subsets (indicated as Intervals 1 and 2 respectively in Fig 1) and treat them as a replication of the same general process.

*Trilobatus sacculifer* generally occurred at higher abundances of up to 38.4% (on average 12.7%) of the entire planktonic Foraminifera assemblage. From *c*.458 kyrs BP the abundance of the species decreased gradually until a local extinction between 439.1 and 438.6 kyrs BP, approximately 500–1000 yrs later than the comparable event in *O*. *universa*. Based on the species’ relative abundance we defined three phases of population size leading to the extinction (Phases 1–3 in Fig 1). Phase 1 with high abundances (24% mean) spans between 457.6 and 451.3 kyrs BP, Phase 2 with medium abundances (10% mean) between 450.7 and 446.8 kyrs BP, and Phase 3 with low abundances (4% mean) between 446.2 and 439.1 kyrs BP. Those phases could be explicitly investigated for morphological developments within the community.

#### Morphotype abundances

In *O*. *universa*, two traditionally distinguished morphotypes (‘*B*. *bilobata*’ and ‘*O*. *suturalis*’) occurred with a mean abundance of 2.4% (Fig 1). ‘*Biorbulina bilobata*’ occurs in marginally higher abundances (on average 1.4%) than ‘*O*. *suturalis*’ (on average 1.0%). The abundance of abnormal morphotypes decreases over time, as shown by a GLM over the entire time period (*p* < 0.001, Nagelkerke-*R*^2^ = 0.22; S1 File). When testing both intervals separately we observe that the decrease in abnormal morphotypes over time is significant for the second interval which leads to local extinction (*p* = 0.002, Nagelkerke-*R*^2^ = 0.22) but not for the first interval (*p* = 0.773, Nagelkerke-*R*^2^ = 0.01). In the *T*. *sacculifer* complex, we find higher abundances of the sacculifer-morphotype of up to 24% broadly coinciding with Phase 1 of the abundance of the species (Fig 1). During early Phase 2, this morphotype decreased rapidly in abundance and, although it never vanished completely within the limits of confidence, never comprised more than 10% of the population from Phase 2 onwards. A constant decline over time is indicated by the GLM (*p* < 0.001, Nagelkerke-*R*^2^ = 0.56; S1 File). This is confirmed by a *z*-test: The abundance of the sacculifer-morphotype differs significantly (*p* < 0.001) between all three Phases, with decreasing mean incidence of that morphotype from Phase 1 (14.7%) over Phase 2 (5.8%) to Phase 3 (2.0%).

### Morphology of *Orbulina universa* across replicated drops in abundance

**Fig 2:**
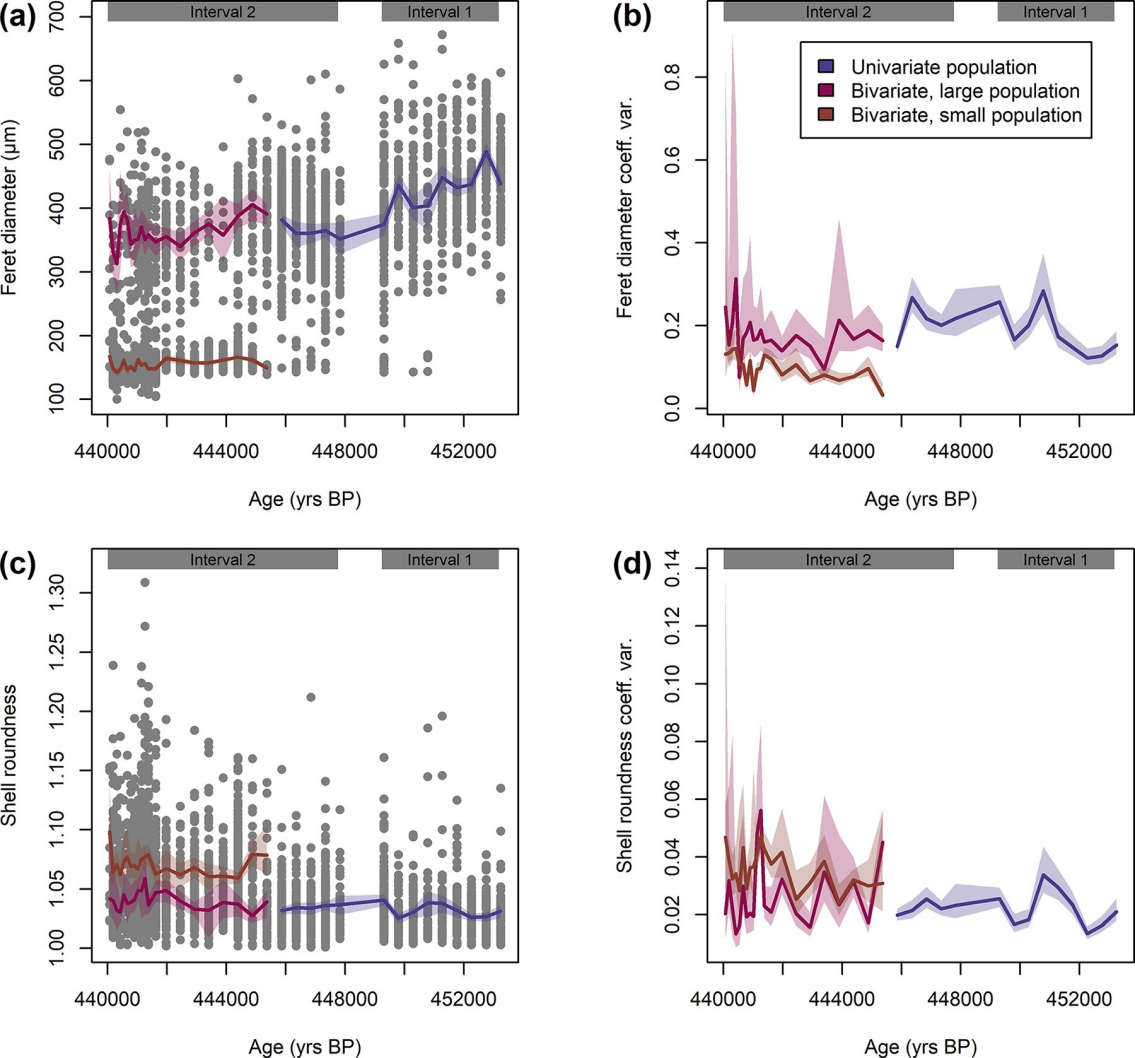
Morphology of *Orbulina universa* from marine isotope stage 12 in the Red Sea. (a) Shell size is showing bimodality after 445.4 kyrs BP, when a small population slowly appeared. (b) The variation in shell size is larger in the large population but generally decreased over time. (c) Shell roundness is rather stable in the large population, and considerably lower in the small population. (d) The variation in shell roundness is considerably higher in the small population but decreased slightly in the larger population toward local extinction. Raw values are plotted as grey dots, mean values as lines, and 95% confidence intervals as shaded areas. The intervals based on species abundance (compare Fig 1) are indicated.

Morphological parameters of *O*. *universa* are presented in Fig 2. The shell size of *O*. *universa* specimens indicates the successive establishment of two populations, as indicated by Hartigan’s dip test and the coefficient of bimodality (S1 File). Until 445.4 kyrs BP only one population with large shells (on average 399 μm) was present. After that, the size distribution reveals the existence of two populations with different sizes. One population shows an average size of 368 μm and can be considered a continuation of the population that was present before the split (Fig 2A). While it differs in size from the larger population before the split on average (Mann–Whitney *U* test, *p* < 0.001) this can be explained by the gradual decrease in size of the larger population over time revealed by robust line fitting (*R*^2^ = 0.08, *p* < 0.001; Fig 2A, S1 File). The second population is significantly smaller (*p* < 0.001), only 155 μm on average, and only occurs with few specimens before the split from *c*.451 kyrs BP onwards. This population shows the same trend of decreasing size toward the local extinction (*R*^2^ = 0.12, *p* < 0.001; Fig 2A, S1 File). The large population became only marginally rarer; it was more abundant before the split, but never disappeared from the sedimentary record until the local extinction. Noteworthy, the coefficient of variation of the larger population was significantly dropping at the splitting point from 0.21 to 0.18 (S1 File). However, the smaller group showed an overall strongly reduced coefficient of variation of only 0.11 (Fig 2B). The final split occurs within Interval 2 and is unrelated to the replicated abundance pattern in *O*. *universa*. Overall, shell size of the assemblage decreased toward the local extinction. When testing for a relationship between shell size and species abundance, a robust line fitting reveals no significant correlation between accumulation rates and shell size for the large (*p* = 0.070) population. For the small population, the regression is significant (*p* < 0.001) but explains very little variation in the data (*R*^2^ = 0.09; S1 File).

To investigate shell roundness in *O*. *universa* we excluded ‘*B*. *bilobata*’ and ‘*O*. *suturalis*’ specimens (*n* = 59 specimens) from the analyses because both deviate significantly from the normal morphology of a terminal shell of that species. Shell roundness shows no indication of bimodality and is constantly decreasing over time according to a robust line fitting (*R*^2^ = 0.04, *p* < 0.001; Fig 2C, S1 File). The latter trend, however, is the result of the smaller population with inherently reduced shell roundness increasing in abundance. A secondary trend of shell roundness being correlated with species abundance exists, but it is much less pronounced (*R*^2^ = −0.01, *p* < 0.001; S1 File). Shell roundness shows a decrease in variation in the large population toward the local extinction (S1 File) but is considerably higher in the small population (Fig 2D). Noteworthy, the major trends in shell size and shell roundness are uncorrelated to the intervals defined by species abundance patterns.

### Morphology of *Trilobatus sacculifer* during a long, continuous extinction event

**Fig 3:**
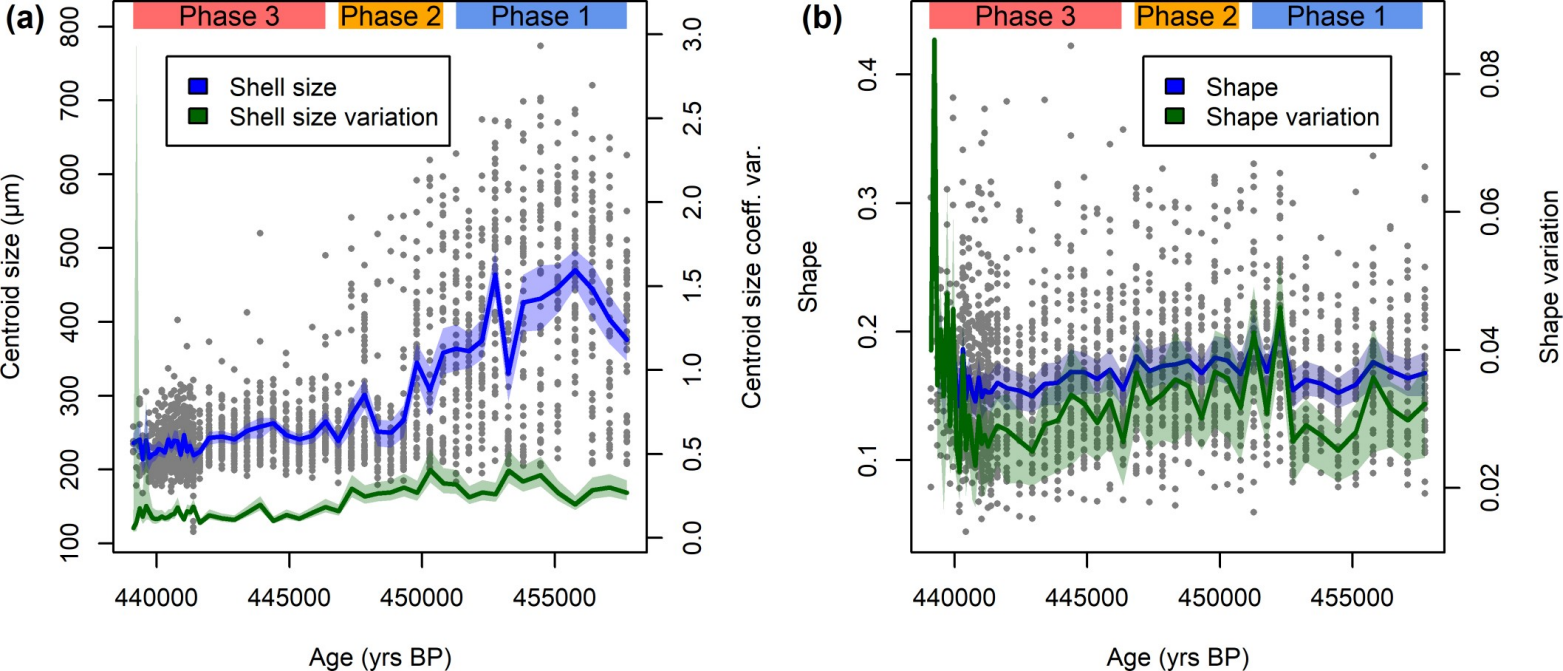
Morphology of *Trilobatus sacculifer* from marine isotope stage 12 in the Red Sea. The three phases defined by abundance (compare Fig 1) are indicated. (a) Shell size and its variation decrease toward the local extinction (compare S1 File). Raw values (grey dots) are plotted alongside the sample mean and coefficient of variation (solid lines) and their 95% confidence intervals (shaded area). (b) Shell shape (Riemannian shape distance from the mean shape) excluding sacculifer-morphotypes (compare S1 File). Raw values (grey dots) are plotted alongside the sample mean and standard deviation (solid lines) including the 95% confidence interval (shaded area).

Morphological parameters of *T*. *sacculifer* are presented in Fig 3. The shell size shows a comparable pattern to the incidence of the sacculifer-morphotype, with larger shells during Phase 1, a rapid size decrease during Phase 2, and small shells during Phase 3 (Fig 3A). Comparing the values within the phases reveals a decrease in mean shell size from Phase 1 (411 μm) over Phase 2 (279 μm) to Phase 3 (237 μm). The differences in size are significant between all groups (*p* < 0.001 for a Kruskal–Wallis test, with all *p* < 0.001 in pairwise Mann–Whitney *U* tests). Moreover, the variation of shell size decreased significantly during Phase 2 (Fig 3A, S1 File). This decrease in shell size and variation is nearly exclusively caused by the lack of large specimens after Phase 2, while the size of the smallest specimens remained rather constant during the entire interval.

Using geometric morphometrics allows a more detailed analysis of the shape of *T*. *sacculifer* shells (Fig 3B). Since the sacculifer-morphotype is a considerably derived morphology, we left it out of the dataset for most analyses (*n* = 136 specimens), but results including the sacculifer-morphotype specimens are presented in S1 File for comparison.

**Fig 4:**
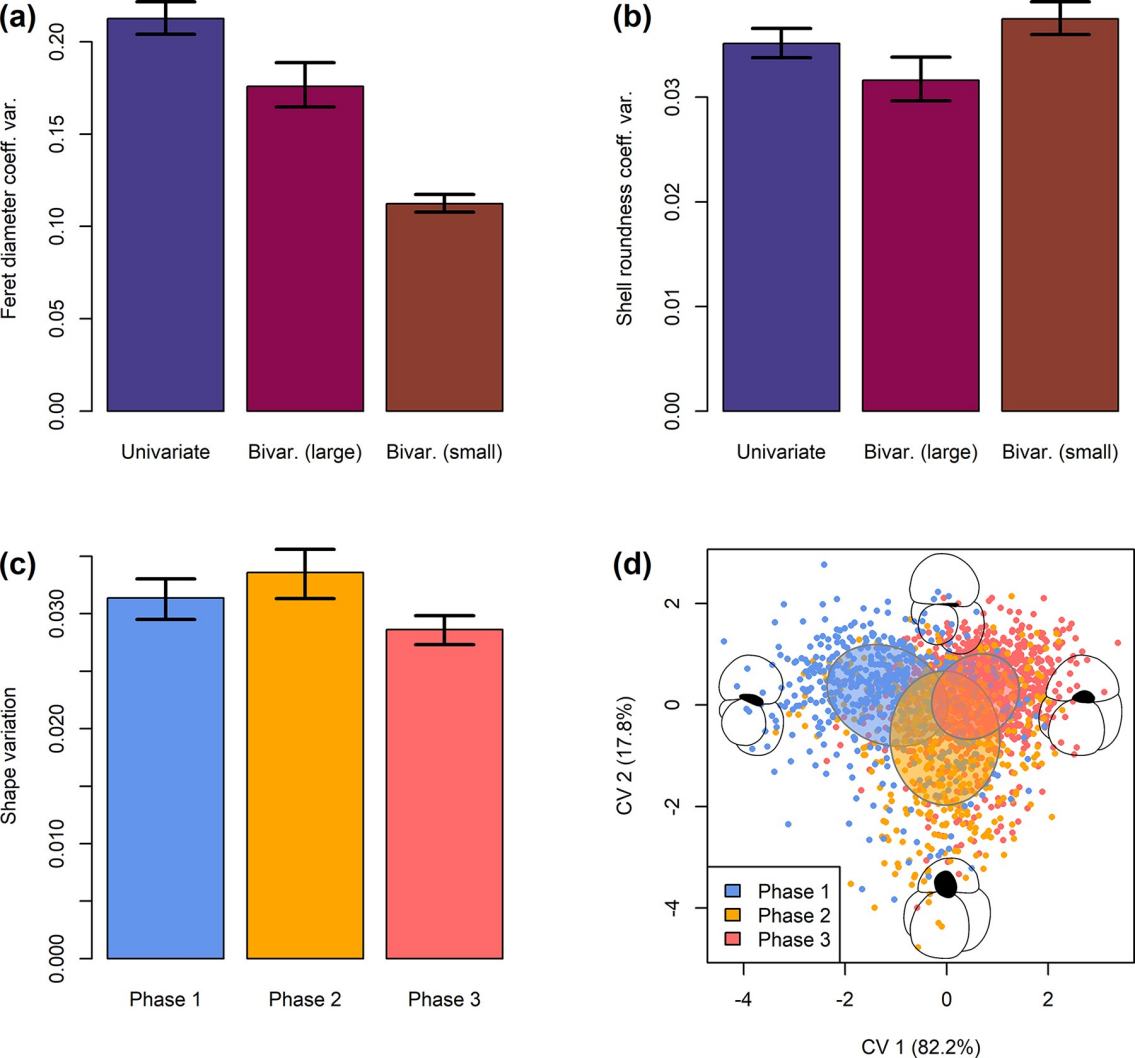
Stabilizing selection in planktonic Foraminifera during marine isotope stage 12 in the Red Sea. (a–b) The variation of shell size and shell roundness (including 95% confidence interval) in the incumbent population of *Orbulina universa* decreased toward local extinction. The small population had an inherently reduced size variation but higher variation of shell roundness. (c) The shape variation (Riemannian shape distance from the mean shape, excluding sacculifer-morphotype) in *Trilobatus sacculifer* decreased significantly toward the local extinction (Phase 3, compare Fig 1). Error bars depict the 95% confidence interval. (d) Canonical variates analysis (CVA) of the shape of *T*. *sacculifer* (excluding sacculifer-morphotype). Points indicate specimens, ellipses indicate the 95% confidence interval of the standard deviation on the centroid, black silhouettes depict the morphology at the extremal points of the canonical variate (CV) 1 and 2 axes, respectively.

The shape of *T*. *sacculifer* specimens between phases differs significantly (NPMANOVA, *p* < 0.001). A *post-hoc* pairwise comparison of shape differences reveals, that the shape of *T*. *sacculifer* specimens differs between all three phases at *p* = 0.002, with a gradual shape change during the last *c*.10,000 yrs before local extinction (Fig 3B). Shape variation showed no trend over time according to a robust line fitting (*R*^2^ = −0.1, *p* = 0.746), but would be significant when including the sacculifer-morphotype (*R*^2^ = 0.1, *p* < 0.001; S1 File). This implies that a large part of the shape variation is linked to the disappearance of the sacculifer-morphotype in the population. Overall, however, there is a clear decreasing trend in shape variation between the end of Phase 1 and close to the end of Phase 3 (S1 File). The variation was rather low during early Phase 1 and seems to have increased very shortly before extinction, but the latter signal is accompanied by large confidence intervals due to the small sample size and should be treated with caution. This complex pattern likely obscures an overall trend, leading to an insignificant regression analysis. When comparing integrated values across the three phases defined by abundance patterns, the data reveal that variation was highest in Phase 2, mediocre in Phase 1, and lowest in Phase 3 (Fig 4C). A *t*-test reveals that variation differences between Phases 1 and 2 are insignificant (*p* = 0.944), but specimens from Phase 3 differ significantly from specimens from both Phase 1 (*p* = 0.010) and Phase 2 (*p* < 0.001). This implies a significant reduction of phenotypic plasticity in the community when exposed to higher stress levels and impeding local extinction.

When analysing the shape change from phase to phase (Fig 4D) it is revealed that from Phase 1 to Phase 2 the terminal chamber became flatter and the aperture was inflated. At the same time the lower part of the shell containing the older chambers became more voluminous, so that there is a general trend towards a smaller terminal chamber in relation to the older chambers. This trend is reversed in Phase 3, with the terminal chamber becoming more inflated and the aperture flattening again. This led to a strong increase in the size of the terminal chamber in regard to the older shell.

## Discussion

### Error analysis

Morphometric data are especially prone to errors, because many things (like the orientation of specimens and digitization of landmarks) are done manually. We therefore analysed our data for the influence of potential error sources, following methods described by Yezerinac, Lougheed [68]. We could not detect any errors of severe size in our data and show a full error discussion in S1 File.

### Morphological stabilization in planktonic Foraminifera

The concept of phenotypic variation [6,69] describes the observed morphological variation among specimens of a population, and is rooted in phenotypic plasticity (the ability of a single genotype to produce several phenotypes). It is thus contrasted to variability, which is the potential of a population to vary within the borders of the genetic encoding [70]. Importantly, phenotypic plasticity can only use pre-existing variability to adapt to environmental changes and is thus limited to a pre-adaptation of the population [71]. A reduction in variability, manifested in reduced morphological variation due to selection for a specific phenotype, is thus a sign of stabilizing selection [4,6,72], where the pre-existing reaction norm of a species is reduced. When in contrast faced with unprecedented environmental change, a population needs to innovate to overcome the new environmental stress by development of new mutations that serve adaptation [11,72]. This is especially important in planktonic Foraminifera, where the existence of (pseudo-)cryptic species can lead to an underestimation of genetic diversity and thus variability of the population. In *T*. *sacculifer* we can rule out that possibility, because despite its large morphospace it only contains one biological species with large variation [42]. In *O*. *universa*, the morphospecies encompasses several biospecies [73], but at present only one genotype is known to occur in the Red Sea [74].

Within our natural experiment, we observe bimodality in shell size coinciding with increasing salinity in the Red Sea in *O*. *universa*. A decrease in size and mean roundness of the incumbent large population is apparent when the species approached its local extinction in the aplanktonic zone. In both parameters, an unbiased random walk pattern best describes the observation (Table 1), implying that the observed morphological change is the result of accumulation of random changes rather than directional selection. The smaller population, however, shows stasis in both parameters without any distinctive trend one way or the other. A reduction in shell size has been proposed to indicate suboptimal environmental conditions [75,76] but also a buoyancy adaptation to increasing salinity in order to be able to stay at an optimum depth in denser water [77]. Since the strong salinity increase in the Red Sea associated with the onset of the aplanktonic zone shifted the local habitat away from the optimum requirements of the incumbent species, it is therefore hard to say whether the trends we observe are the result of abiotic or biotic forcing. However, if the observed morphology change would be a purely biotic stress response, assuming that abundance is a useful proxy for the suitability of the environment [78], we would principally expect to see a comparable development in Interval 1 as in Interval 2. In contrast to that assumption, neither shell size nor shell roundness show any signs of a deviation associated with the first abundance drop at the end of Interval 1. We further observe that shell size and shell roundness are significantly different between both intervals at *p* < 0.001, which is not what one would expect if they would show a comparable internal pattern. We therefore argue that the observed size trend is likely an adaptation either of buoyancy to remain in a favourable water depth when the salinity and thus density of the ambient sea water increases [77] or dictated by the lower nutrient availability in a disrupted ecosystem under higher salinities, that does not allow precipitation of excessive amounts of calcite [40].

**Table 1:** Results of phyletic evolution models of planktonic Foraminifera in the Red Sea during marine isotope stage 12.

Parameter	Phyletic model	AIC_c_	Akaike weight
***Orbulina universa***			
Shell size total	GRW	377.84	0.306
	URW	376.20	0.694
	Stasis	425.81	0.000
Shell size large pop.	GRW	322.40	0.328
	URW	320.96	0.672
	Stasis	357.67	0.000
Shell size small pop.	GRW	157.96	0.014
	URW	155.22	0.055
	Stasis	149.58	0.931
Shell roundness total	GRW	−246.40	0.371
	URW	−247.45	0.629
	Stasis	−192.50	0.000
Shell round. large pop.	GRW	−257.97	0.200
	URW	−260.72	0.791
	Stasis	−251.90	0.010
Shell round. small pop.	GRW	−131.90	0.034
	URW	−135.27	0.185
	Stasis	−138.16	0.781
***Trilobatus sacculifer***			
Shell size	GRW	506.03	0.311
	URW	504.44	0.689
	Stasis	603.25	0.000
Shell shape	GRW	−291.21	0.133
	URW	−293.50	0.416
	Stasis	−293.67	0.452

GRW: Generalized random walk, URW: Unbiased random walk, shell shape values of *T*. *sacculifer* excluding the sacculifer-morphotype

We interestingly observe a reduction in the abundance of abnormal morphotypes with increasing stress levels, which also fits with the observation of overall reduction of morphological variation. Abnormal morphotypes in *O*. *universa* where suggested to either indicate unfavourable environmental conditions [39] or favourable environmental conditions with high nutrient supply [40,79]. Our observations support the latter hypothesis, because the incidence of abnormal morphotypes in our samples decreases with increasing stress levels and might well be correlated with higher productivity in a more normal-marine setting earlier during the study interval (where most probably the entire community was more healthy, generating more nutrients across the trophic network). This is in line with observations by Weinkauf, Moller [12], where the increase in abnormal morphotypes toward the sapropel formation may coincide with higher nutrient availability due to increased continental runoff.

The observation of an incumbent large population which shows a morphological development characteristic for stress situations and the splitting of a small population showing stasis would be consistent with two possible end-member scenarios: (1) A monospecific *O*. *universa* population present at the time in the Red Sea shows signs of disruptive selection as a result of exposure to a suboptimal environment [5], or (2) a new population comprising a different biospecies with different morphology is introduced into the Red Sea once environmental conditions become more suitable.

To investigate those scenarios, we applied a Kendall rank-order correlation between individual shell size and shell roundness and found that they are significantly correlated (*ρ* = −0.321, *p* < 0.001; S1 File). This implies that smaller individuals also tend to have less round shells. To test this, we divided the population into two subsets at the local minimum of the size distribution (238.6 μm) and tested the shell roundness in both subgroups against each other with a Mann–Whitney *U* test. The test confirms that the larger population produced shells that are on average significantly (*p* < 0.001) rounder (mean = 1.03) than the smaller subpopulation (mean = 1.07). The fact that the maximum shell roundness is practically 1.00 in both populations shows, that the observed trend is neither cause by a biological inability of shells to grow very round below a certain size nor the result of a lower precision of shell roundness determination in smaller shells. Comparing the 95% confidence interval of the coefficient of variation shows a significantly higher variation of shell roundness in the population with smaller shells (0.036–0.039) than in the population with larger shells (0.020–0.022; Fig 4). Noteworthy, shell roundness variation did not meaningfully increase over time in either population, which shows the variation as inherent to the respective population (Fig 2D). It is also possible that shell roundness and shell size are biologically integrated in *O*. *universa*, so that a change in one parameter necessitates a certain change in the other value [80]. This would be an alternative explanation for the observed correlation between individual shell size and shell roundness, but the very low covariance between both parameters (−0.008) together with the fact that small specimens realize the complete range of possible deviations from sphericity principally argues against that hypothesis. The mechanistical advantage of less round shells must remain speculative but may increase the volume and surface area of the shell in comparison to a sphere of the same size to allow the storage of more photosymbionts and higher photosynthetic activity to offset detrimental effects by the increased salinity.

Our observations do not allow us to decide for one of the two potential hypotheses. The Red Sea was possibly invaded by a population with smaller, more variably shaped shells from approximately 445.4 kyrs BP onwards, which increasingly established its presence at the expense of the incumbent population due to their better adaptation to the local environment. Should this be true, we would find here the first example where different *O*. *universa* biospecies could be distinguished on the basis of relatively easily obtainable morphological characters [81] and also the first example of more than one *O*. *universa* biospecies occurring in the Red Sea [74]. Furthermore, it would provide evidence for different ecological preferences among those biospecies, which facilitates competitive exclusion due to increasing stress levels [71]. However, we would argue that this explanation is unlikely on the basis of other assumptions. (1) The potentially invading species would likely had had to be of Indian Ocean origin. Within an environmental setting of increasing salinity, it is hard to perceive that any species from the open-marine Indian Ocean would have a selective advantage over a native species from the Red Sea, that should be better adapted to high salinities [82]. (2) A trend of decreasing shell size with increasing salinity in *O*. *universa* was already observed by Haenel [77], and was there attributed to buoyancy requirements, although Spero [40] saw a closer relation to nutrient availability due to the energy requirements to build larger shells. This makes it reasonable that the observed size change is indeed an adaptive response by part of the population. (3) The splitting in two size populations as well as the increased variation in roundness in the smaller population both imply disruptive selection [83]. We thus favour the explanation that the incumbent *O*. *universa* population underwent disruptive selection as a result of environmental stress, associated with drastically increasing salinity levels in the Red Sea. This would replicate results obtained on a Mediterranean population that was exposed to higher stress levels in relationship with the onset of sapropel deposition [12], and would lend valuable evidence to the assumption, that certain morphological changes can be universally interpreted as indicator of environmental stress, at least when the same species are considered. The small subspecies would in that scenario be the better adapted result of accumulated random change [84], and would fall into a state of evolutionary stasis once apparently optimal adaptation is reached. This conforms with the proven development of stasis in extreme environments [85], while both populations can co-exist as long as an equilibrium point exists along the Lotka–Volterra isoclines [71]. Divergence without speciation is often observed in nature, and new character traits that are encompassed in the variability of the population can be fixed, once a diverging subpopulation occupies a new niche [11]. In the present case, the size difference in the two *O*. *universa* populations points toward a different in the depth habitat due to differential buoyancy [77]. While thus disruptive selective pressure in *O*. *universa* may have led to the development of two populations, each individual population shows signs of stabilizing selection in its shell size parameter. It is very likely that the emerging new population is not the result of an evolutionary innovation, but the reactivation of pre-existing genetic variability in part of the population. This is evidenced by the rapid emergence via unbiased (in contrast to general) random walk and subsequent stasis of the small population as well as its ultimate failure to cope with the environmental change, which is not what would be expected under a scenario of dynamic evolution [72].

*Trilobatus sacculifer* draws a very similar picture in the form of decreasing variation in its shell size and shape over prolonged time spans before local extinction (Figs 3 and 4). In shell size this decrease complies with the unbiased random walk model, while for shell shape the distinction between unbiased random walk and stasis is nearly impossible (Table 1).

The size decrease may itself be a signal for decreasing environmental suitability for the species [75,76], governed by the same rules as in *O*. *universa* (buoyancy and nutrient availability described above). The decrease in variation is a clear signal for stabilizing selection inducing microenvironmental canalization (the propensity to maintain a stable narrow phenotype in spite of environmental variation) [6,86]. It is interesting, that stabilization occurs toward Phase 3, which must have been clearly environmentally suboptimal for *T*. *sacculifer* (drop in abundance, drastic increase in salinity). This could indicate either a stabilization of the regional environment, allowing selection for an optimal trait [4], or a rapidly changing environment enforcing fluctuating selection that benefits a stable phenotype in the long run [87,88]. The slight increase of variation between Phases 1 and 2 may be an indicator for the second explanation, and indicate a Baldwin effect [89], i.e. a phase of increased plasticity that allows adaptation to a new environment and evolutionary fixation afterwards. The reduction in the abundance of the sacculifer-morphotype after Phase 1 could be another manifestation of the stabilizing selection, but it could also have resulted from an inability of *T*. *sacculifer* shells to build an asymmetric chamber below a certain chamber size threshold. In the latter case, the drop in the abundance of the sacculifer-morphotype would result from the shell size decrease and not necessarily be a signal for stabilizing selection.

We observe certain morphological trends in *T*. *sacculifer* over time, as shown in Fig 4, which can be interpreted as adaptive for a changing environment that gradually becomes less suitable for the population. During Phase 1, shells show more inflated and larger terminal chambers. This is reminiscent of the sacculifer-morphotype, which was left out of the CVA due to its strongly derived morphology. This phenotype is often assumed to be more abundant under lower stress levels [90] but has already earlier been shown to be correlated with larger shell sizes [91], which may result from a presumably higher nutrient availability in the more open marine conditions earlier in the studied interval. During Phase 2, coinciding with a first strong drop in sea level and thus salinity increase (Fig 1), the abundance of the sacculifer-morphotype and the shell size decreased, while the size of the terminal chamber decreased as well, indicating a trend towards Kummerforms that are often associated with unfavourable environmental conditions [92]. Finally, in Phase 3 the shell size decreased further while the terminal chamber became larger again in relative terms. The latter trend could be necessitated by the small shell size, so that the terminal chamber had to become relatively larger again to provide enough space for gametogenesis. This phase is also characterized by a canalization peak, probably induced by an environment that was so unstable and unfavourable for the *T*. *sacculifer* population that any deviation from a very narrow morphotype at the edge of the species variability would drastically decrease survival rates and fitness. The entire investigated timespan can possibly be interpreted as follows: During Phase 1 an incumbent population of *T*. *sacculifer* was well adapted to the high but not excessive salinity levels in the Red Sea during MIS 12. During Phase 2, salinity levels started to rise, and induced a selection that via accumulation of random change led to a modification of the phenotype [84]. In this phase, a Baldwin effect set in, increasing the variation of shape and thus buying the population time until further adaptation could settle in [89]. During Phase 3, the adaptation was as complete as it could possibly become within the variability of the species, and the successful morphologies became fixed via canalization [6,11,93]. It is interesting to note that the trend towards smaller shells that was observed in *O*. *universa* is also present in *T*. *sacculifer*. This indicates that probably buoyancy problems due to the increasing water salinity were responsible for this adaptive trend.

*Trilobatus sacculifer* interestingly shows a noteworthy increase in shape variation very shortly before extinction, which to some degree seems to contradict the stabilizing trend that was prevalent until then. This trend could be interpreted in two ways. It was either a renewed, and in this case unsuccessful, Baldwin effect [89]. Alternatively, it may be the ultimate result of drastically increased fluctuating asymmetry within a population exposed to ever growing environmental change reaching the limits of its variability [2,3]. In both cases, it indicates a population under extensive stress levels and close to its collapse in absence of successful adaptation.

### Planktonic foraminiferal adaptability

In our study we analysed the adaptive patterns of two species of planktonic Foraminifera that were exposed to increasing levels of salinity stress. Interestingly, the observations in both species coincide very well. Both species ultimately show signs of stabilizing selection in both shell morphology (Fig 4) and the decreasing incidence of more derived phenotypes (Fig 1). They already become extinct at water salinities which other protist groups still tolerate [25,94–96], showing that the environmental change was not so strong that an adaptation toward it would be biologically infeasible. Both species showed processes that are well in line with adaptive patterns that use pre-existing variability to cope with environmental stress and did not seem able to innovate to overcome the stress levels [6,11,72,86,97]. This is despite the fact that planktonic Foraminifera have very short reproduction cycles of only 4–6 weeks [98], which makes evolutionary processes fast enough that the time encompassed in this natural experiment would have been sufficient for new traits to emerge to adapt to the environmental change [99]. These observations at least hold true as far as adaptations of the shell morphology are concerned. We note that potential adaptive processes on the cellular level may have gone unnoticed due to their lack of fossil preservation. However, foraminiferal cell organelle structures are still only vaguely understood [100,101], so that the interpretation of such changes would in any case be highly speculative and ultimately failed to ensure the survival of the species as well if they indeed occurred.

It therefore seems that at least these two species of planktonic Foraminifera primarily adapt to changing environments through their innate variability, and show a low tendency toward innovation (i.e. a low evolvability [72]). This means that these taxa would be heavily influenced by the exposition to environments outside their pre-adapted range and would prefer stress avoidance (i.e. migration) over innovative adaptation. If this pattern is prevalent amongst all planktonic Foraminifera, it could explain why the global assemblages faithfully tracked climate zone shifts during the Quaternary glacial–interglacial cycles [13,102,103] rather than going through phases of intense radiation.

This study analysed only two species in a limited stress scenario, and further analyses are strictly required to confirm the reduced evolvability in planktonic Foraminifera, although other studies point toward similar conclusions [12,22]. If this lack of evolvability should be confirmed on a larger scale across planktonic Foraminifera however, it would make the entire group vulnerable to unprecedented environmental change scenarios. Given the capital role this taxon plays in marine carbonate sequestration and the carbon cycle [14], this could be a major factor in future climate change scenarios. Due to the feedback between the production of biomass (which removes atmospheric CO_2_) and carbonate sequestration (which releases CO_2_ into the atmosphere) [104,105], the reaction of the marine microplankton could modify climate change scenarios either way depending on the ratio between biomass (population and cell sizes) and carbonate production (shell size and calcification). We therefore advocate for more work in this field to reach a better understanding of planktonic foraminiferal adaptive capabilities and modes of adaptation to environmental protrusions.

## Supporting information

S1 File Morphometric data extraction, additional results, error discussion. (PDF)
