# Supplementary material for: An extinction event in planktonic Foraminifera preceded by stabilizing selection
Source: PLoS One. 2019 Oct 14;14(10):e0223490. doi: 10.1371/journal.pone.0223490 (PMC6791547; doi:10.1371/journal.pone.0223490)
Supplement: S1 File — (PDF) [file pone.0223490.s001.pdf]

Electronic supplements to: 'An extinction event in planktonic Foraminifera preceded by stabilizing selection'

PLoS ONE

Manuel F. G. Weinkauf[1,2,3], *Fabian G. W. Bonitz*[1,a], *Rossana Martini*[3], *and Michal Kučera*[2]

[1]Department of Geosciences, Eberhard–Karls Universität Tübingen, Tübingen, Germany; [2]MARUM, Universität Bremen, Bremen, Germany; [3]Department of Earth Sciences, Université de Genève, Genève, Switzerland; [a]*Current address:* Norwegian Research Centre, Bjerknes Centre for Climate Research, Bergen, Norway

Correspondence to: Manuel F. G. Weinkauf (Manuel.Weinkauf@unige.ch)

1 Material and methods details

1.1 Morphological data extraction

Table S1. Description of landmarks used for *Trilobatus sacculifer* specimens from marine isotope stage 12 in the Red Sea in apertural standard orientation (compare Fig. S1) and their associated landmark type after Bookstein [1].

Landmark	Description	Type
1	Leftmost point of aperture	III
2	Topmost point of aperture in middle part (point of maximum curvature)	II
3	Rightmost point of aperture	III
4	Trisection between aperture, second-youngest, and third-youngest chamber	I
5	Lowermost point of aperture in middle part	III
6	Left intersection between youngest chamber and older shell	I
7	Left point of maximum curvature of youngest chamber	II
8	Topmost point of youngest chamber	III
9	Right point of maximum curvature of youngest chamber	II
10	Right intersection between youngest chamber and older shell	I
11	Intersection between third-oldest chamber and older shell	I
12	Intersection between second-oldest and third-oldest chamber	I

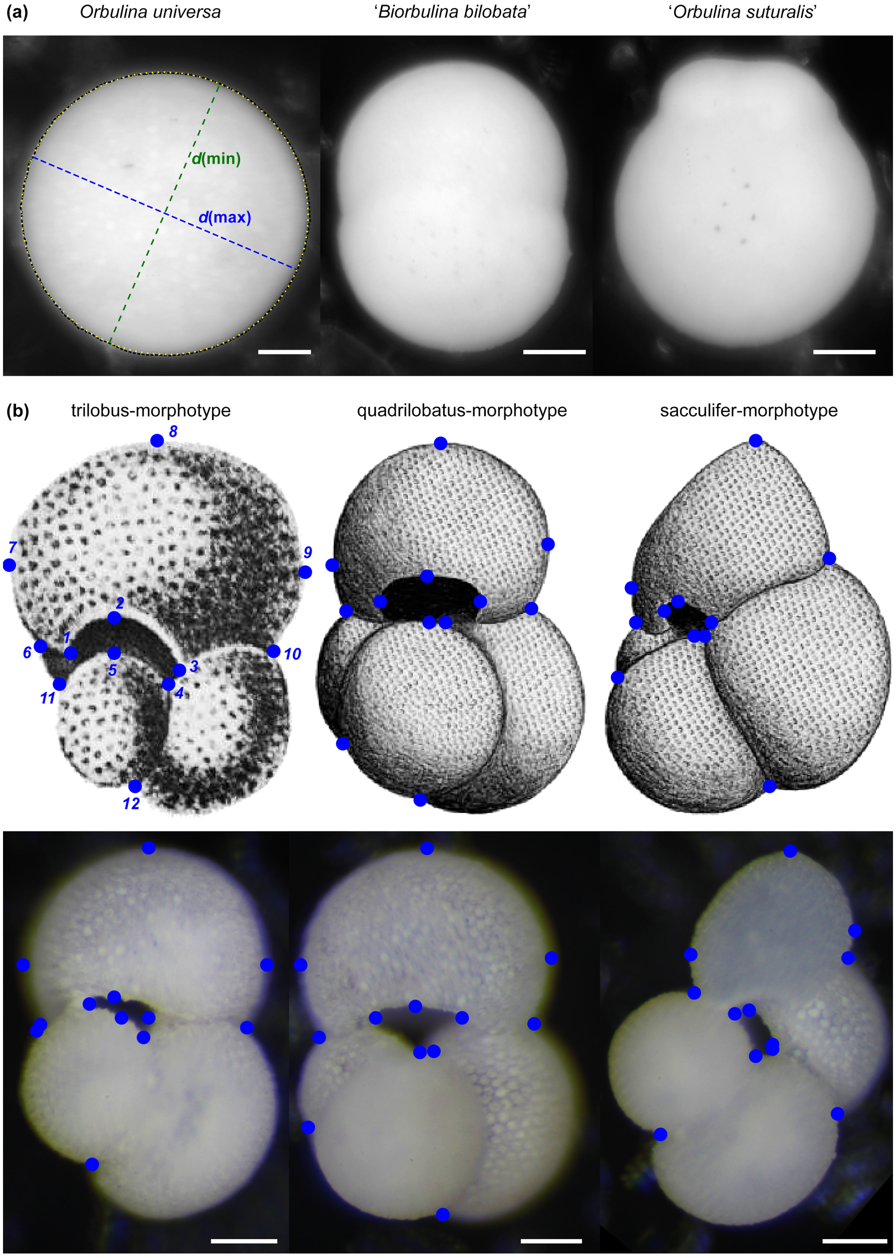

Figure S1. Depiction of species and measurements. (a) Measurements and morphotypes in *Orbulina universa*. Shell size was extracted as Feret diameter (not shown) according to the raw outline of the shell (black, dashed line). Shell roundness was calculated as ratio between the longest (d(max)) and shortest (d(min)) axis of an ellipse fitted to the outline (yellow, dotted line). The incidence of the ecophenotypes '*Biorbulina bilobata*' and '*Orbulina suturalis*' was counted manually. (b) In *Trilobatus sacculifer*, three morphotypes were distinguished, which are shown here in apertural standard orientation. Twelve landmarks were extracted for morphometric analyses (indicated as blue dots and exemplarily numbered in the drawing of the trilobus-morphotype, compare Table S1). The upper row shows type specimen drawings of the three morphotypes: trilobus-morphotype from Reuss [2], quadrilobatus- and sacculifer-morphotypes from Banner, Blow [3]. The lower row shows corresponding light microscopy photographs from the studied samples. Scale bars for all light microscopy images equal 100 µm.

2 Additional results

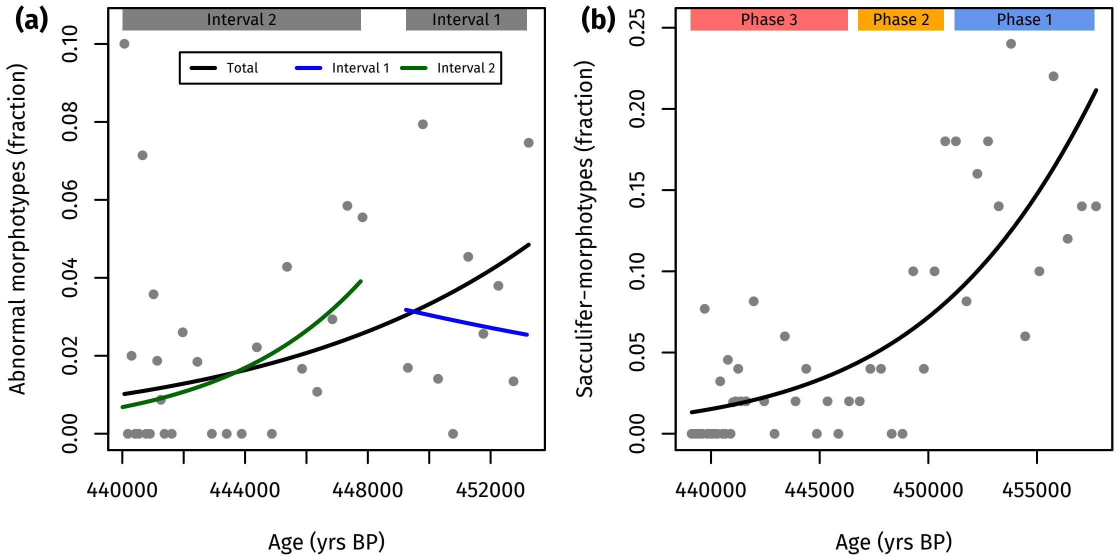

	O. universa (total)	*O. universa* (Interval 1)	*O. universa* (Interval 2)	*T. sacculifer*
Nagelkerke-R^2	0.22	0.01	0.22	0.56
p-value	<0.001	0.773	0.002	<0.001

Figure S2. Generalized linear models (binomial distribution, logit as link-function) [4], of (a) the incidence of abnormal morphotypes in *Orbulina universa* and (b) the incidence of sacculifer-morphotypes in *Trilobatus sacculifer* from marine isotope stage 12 in the Red Sea. The intervals for *O. universa* and phases for *T. sacculifer* based on abundance patterns are indicated.

Table S2. Test for bimodality in shell size and shell roundness of *Orbulina universa* from marine isotope stage 12 in the Red Sea. Shown is the coefficient of bimodality after Ellison [5] and *p*-values of Hartigan's dip test [6] (compare Fig. S3).

		Shell size		Shell roundness	
Age (yrs BP)	Depth in core (cm)	Coeff. bimodality	*p*-value	Coeff. bimodality	*p*-value
440 068.01	1436.25	1.78	0.503	1.41	0.442
440 186.99	1436.75	0.85	0.915	0.54	0.749
440 305.97	1437.25	0.8	0.981	0.48	0.946
440 424.95	1437.75	0.85	0.528	0.49	0.546
440 543.94	1438.25	2.57	0.034	0.78	0.966
440 662.92	1438.75	0.88	0.216	0.51	0.204
440 781.9	1439.25	0.89	0.795	0.54	0.59
440 900.88	1439.75	0.85	0.819	0.46	0.48
441 019.86	1440.25	0.52	0	0.64	0.634
441 138.84	1440.75	0.86	0.492	0.51	0.566
441 257.82	1441.25	0.82	0.966	0.55	0.987
441 376.81	1441.75	0.87	0.383	0.56	0.689
441 614.77	1442.75	0.84	0.969	0.57	0.678
441 971.71	1444.25	0.68	0	0.51	0.722
442 447.64	1446.25	0.72	0.09	0.48	0.041
442 923.56	1448.25	0.88	0.018	0.45	0.789
443 401.5	1450.25	0.94	0.355	0.63	0.974
443 893.51	1452.25	0.85	0.955	0.37	0.921
444 385.52	1454.25	0.86	0.087	0.53	0.387
444 877.53	1456.25	0.82	0.009	0.52	0.607
445 369.54	1458.25	0.62	0.04	0.63	0.209
445 861.55	1460.25	0.35	0.994	0.41	0.846
446 353.56	1462.25	0.65	0.179	0.55	0.676
446 845.57	1464.25	0.31	0.818	0.52	0.471
447 337.58	1466.25	0.25	0.917	0.5	0.559
447 829.59	1468.25	0.33	0.931	0.59	0.964
449 305.62	1474.25	0.4	0.603	0.54	0.273
449 797.63	1476.25	0.34	0.944	0.55	0.989
450 289.64	1478.25	0.24	0.885	0.47	0.903
450 781.65	1480.25	0.55	0.938	0.74	0.847
451 273.66	1482.25	0.33	0.964	0.68	0.544
451 765.67	1484.25	0.45	0.944	0.62	0.835
452 257.68	1486.25	0.31	0.414	0.47	0.846
452 749.69	1488.25	0.41	0.944	0.58	0.505
453 241.69	1490.25	0.33	0.771	0.55	0.981

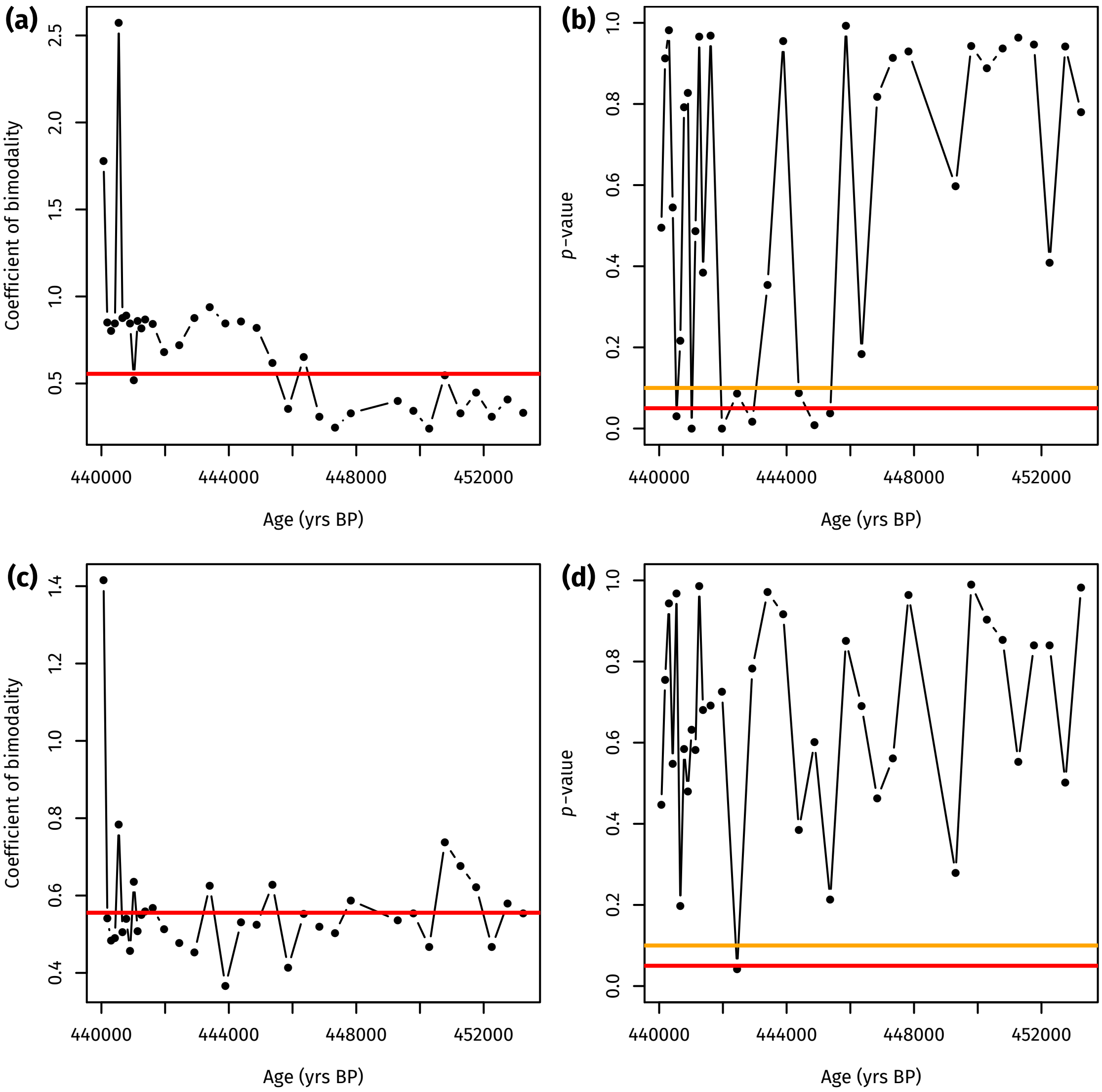

Figure S3. Test for bimodality in shell size (a, b) and shell roundness (c, d) of *Orbulina universa* from marine isotope stage 12 in the Red Sea. Shown is the coefficient of bimodality after Ellison [5] (a, c), where a value larger $\frac{5}{9}$ (red line) indicates bimodality, and results of Hartigan's dip test [6] (b, d), with indications of the $q = 0.05$ (red line) and $q = 0.10$ (orange line) confidence level. Both metrics indicate persistent bimodality in the shell size of the *O. universa* assemblage after 445.4 kyrs BP, but none in shell roundness values (compare Table S2).

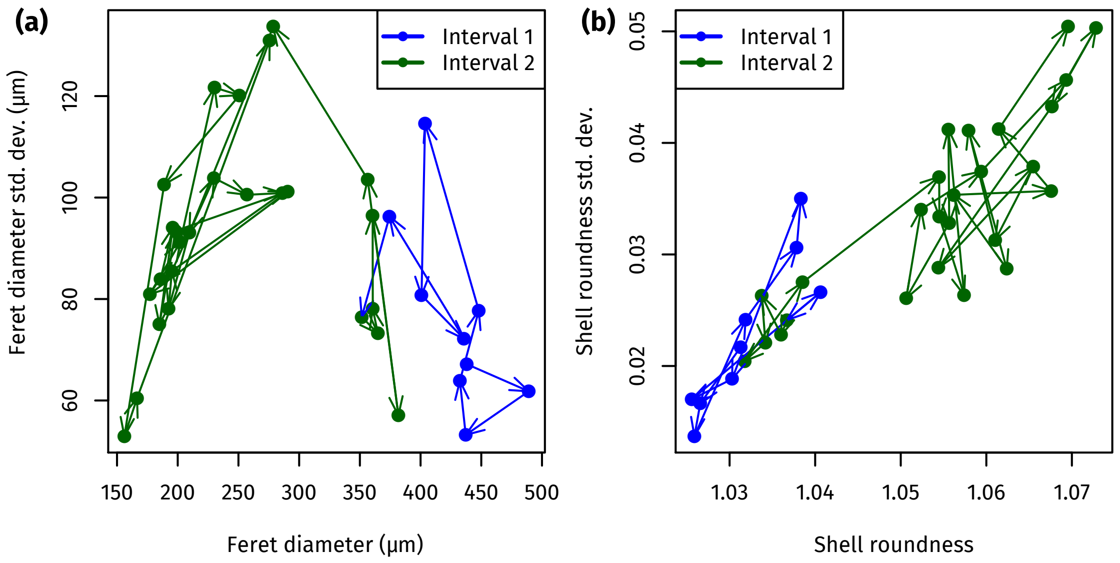

Figure S4. Morphology of *Orbulina universa* from marine isotope stage 12 in the Red Sea. Cross-plots of the entire population, where mean values per sample (points) are connected by arrows in temporal order. (a) Shell size decreases over time but shell size variation remains rather constant. (b) Shell roundness variation increases due to the increase in abundance of the more variable small population.

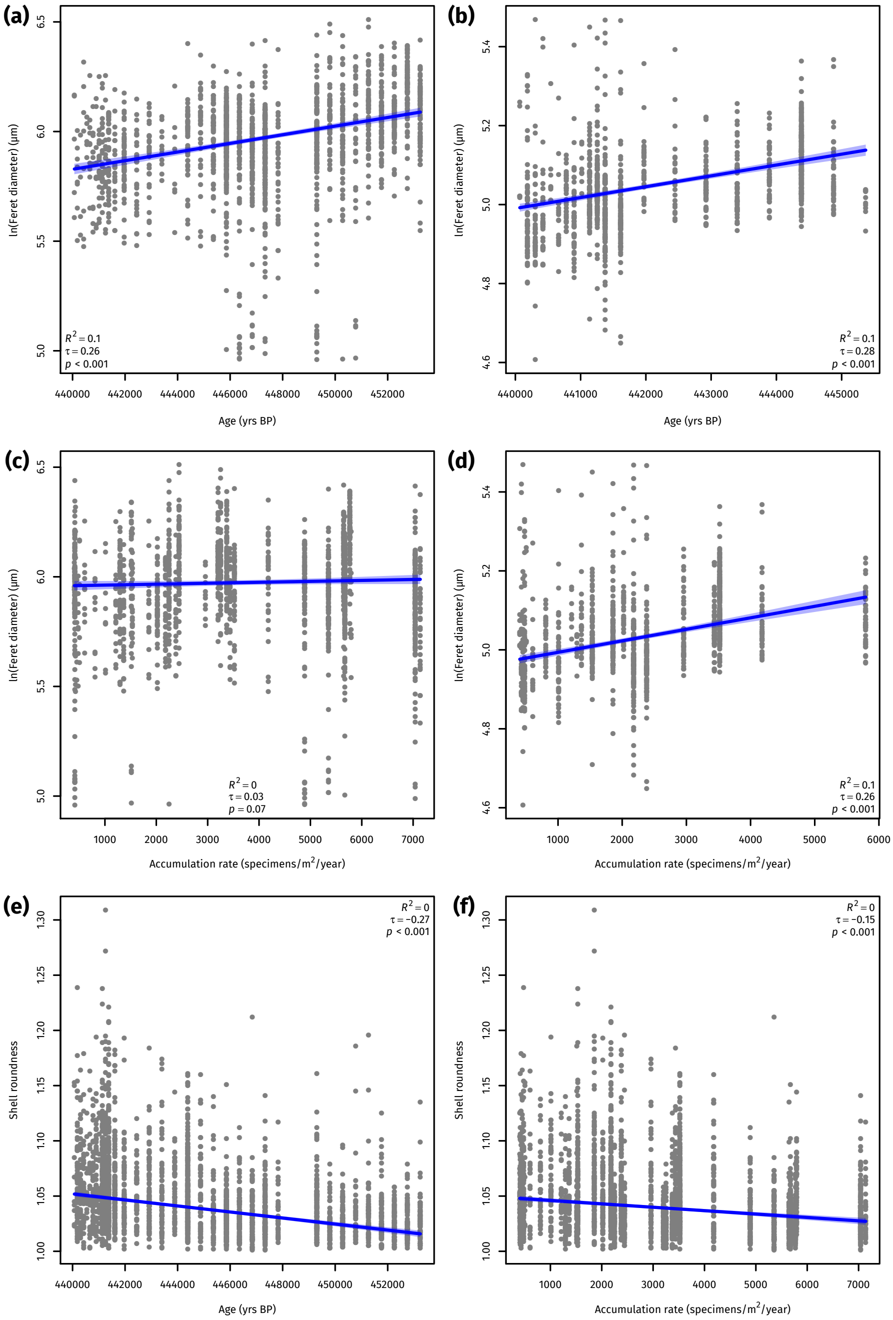

Figure S5. Shell size and roundness trends over time and with species abundance in *Orbulina universa* from marine isotope stage 12 in the Red Sea, using Kendall–Theil robust line fitting [7–9]. (a, c) Shell size for the large population, that is present all the time. (b, d) Shell size for the small population, that appears at 445.4 kyrs BP. (e, f) Shell roundness for the entire population. The 95 % confidence interval of the regression is shown as shaded area. Shell sizes were $\log_e$-transformed prior to analysis.

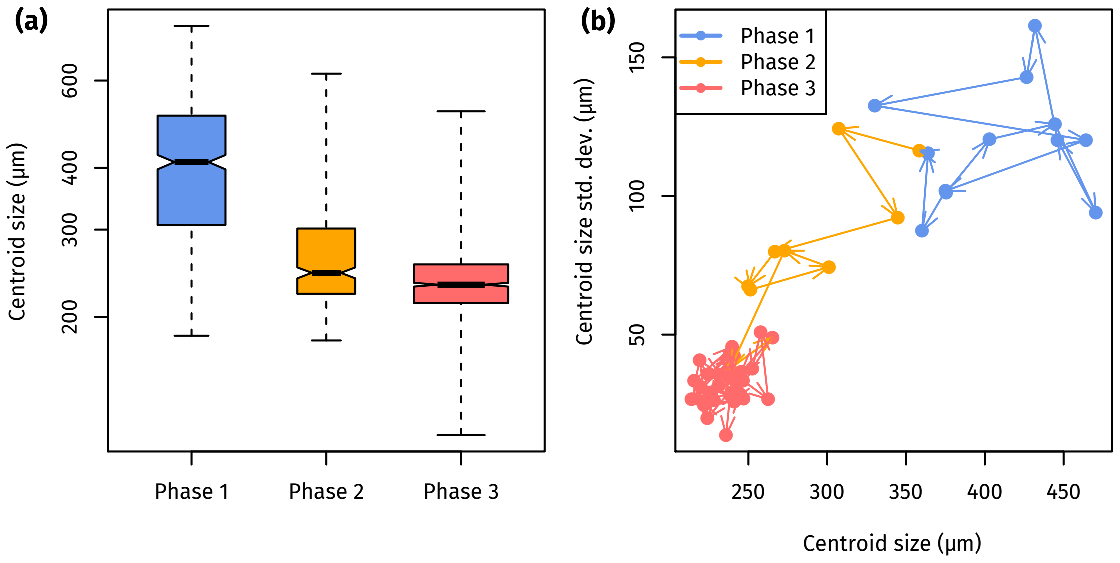

Figure S6. Shell morphology of *Trilobatus sacculifer* through three phases defined by species abundance from marine isotope stage 12 in the Red Sea. (a) Boxplot of shell sizes. Box width is scaled to the number of observations per group, the thick line indicates the median, the boxes extend to the interquartile range, the whiskers cover the entire range of observations. Note the log-scaling of the y-axis. (b) Cross-plot, where mean values per sample (points) are connected by arrows in temporal order.

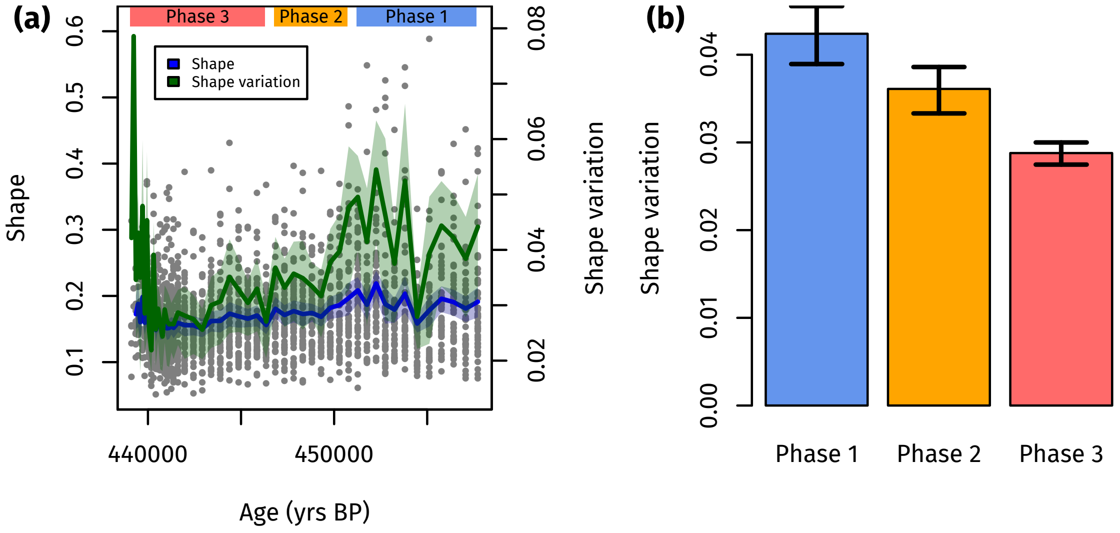

Figure S7. Shape of *Trilobatus sacculifer* from marine isotope stage 12 in the Red Sea, including sacculifer-morphotypes. (a) The shape (Riemannian shape distance) of the shells changed toward the local extinction and its variation dropped. Raw values (grey dots) are plotted alongside the sample mean and variation (solid lines). The three phases defined by abundance are indicated. (b) The shape variation (variance of the Riemannian shape distance from the grand mean) significantly decreased from Phase 1 to Phase 3. The shape between abundance groups differs significantly (NPMANOVA [10], $p < 0.001$), with all pairwise differences being significant ($p = 0.002$).

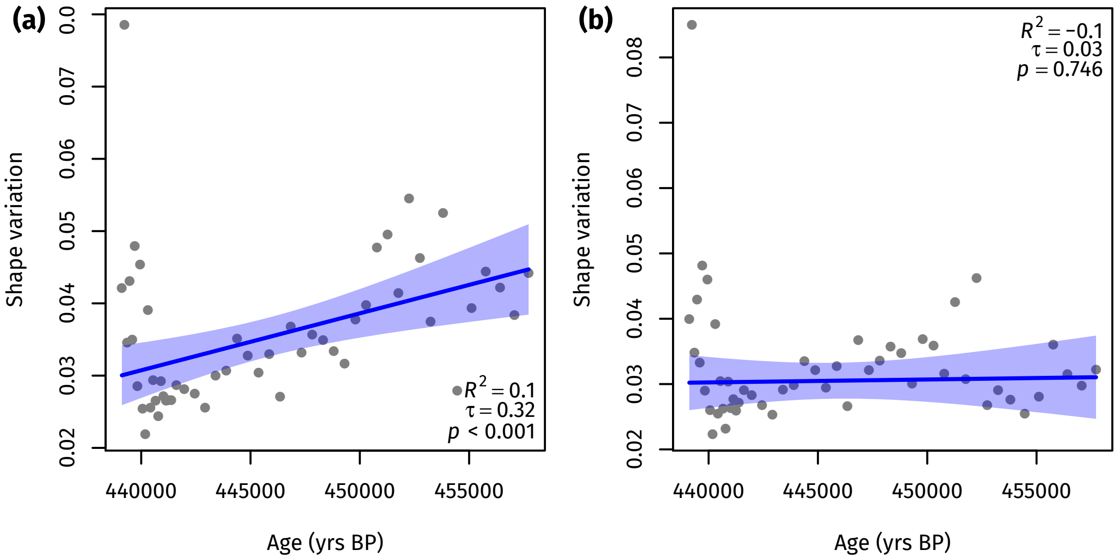

Figure S8. Shape variation of *Trilobatus sacculifer* from marine isotope stage 12 in the Red Sea, using Kendall–Theil robust line fitting [7–9]. (a) When including the sacculifer-morphotype, the shape variation decreased over time. (b) This seems to be mainly related to the extinction of the sacculifer-morphotype, because this trend disappears when excluding this morphotype. The 95 % confidence interval of the regression is shown as shaded area.

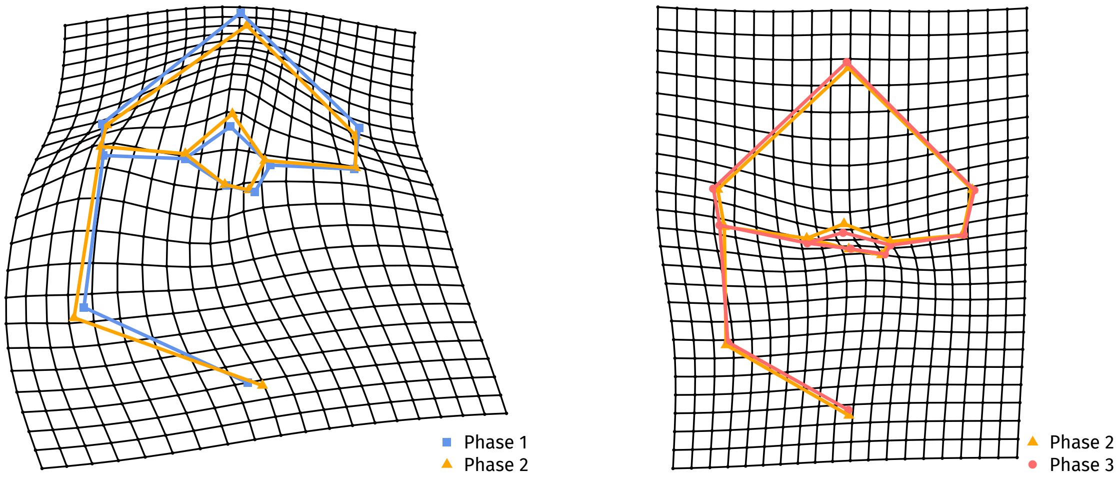

Figure S9. Thin plate splines of the deformation of *Trilobatus sacculifer* specimens from marine isotope stage 12 in the Red Sea between three phases defined by relative abundances.

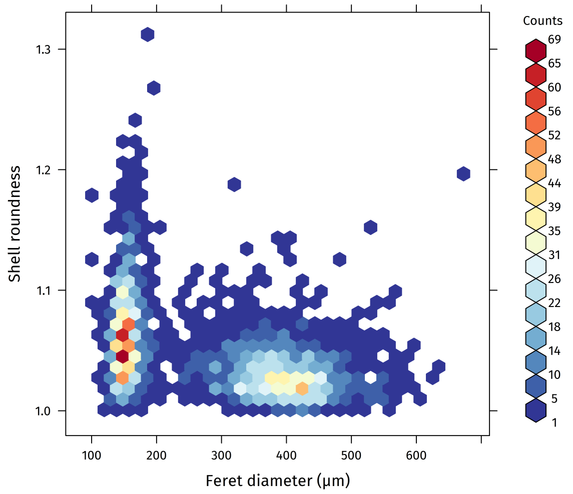

Figure S10. Correlation between shell size (as Feret diameter) and shell roundness in *Orbulina universa* during marine isotope stage 12 in the Red Sea. The population shows clear bimodality in shell size. Smaller specimens show a significantly higher variation of roundness than larger specimens.

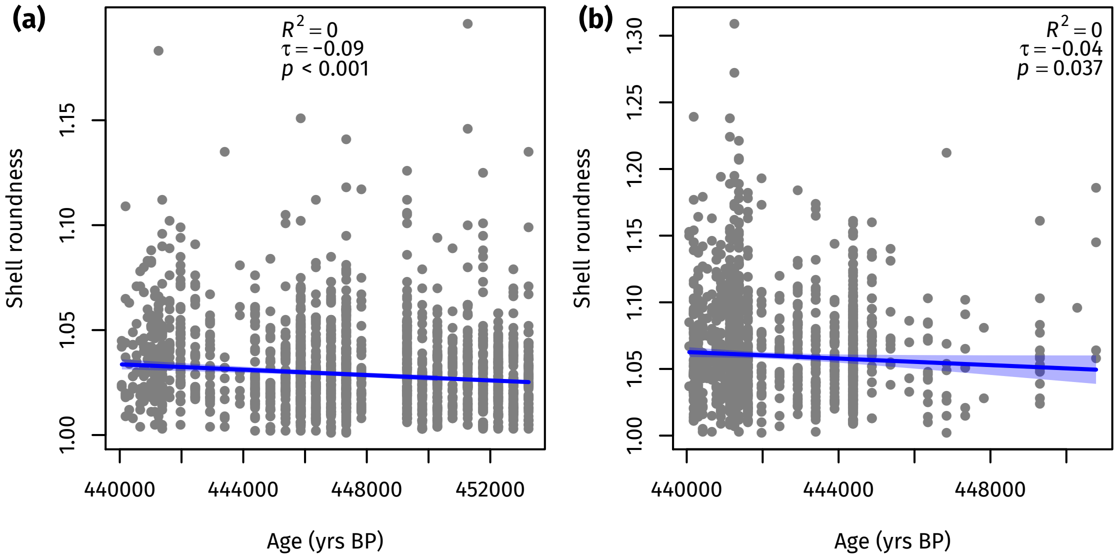

Figure S11. Shell roundness trend in the large (a) and small (b) population of *Orbulina universa* from marine isotope stage 12 in the Red Sea. The regression is significant for both populations but explains practically no variation in the data. The 95 % confidence interval of the regression is shown as shaded area.

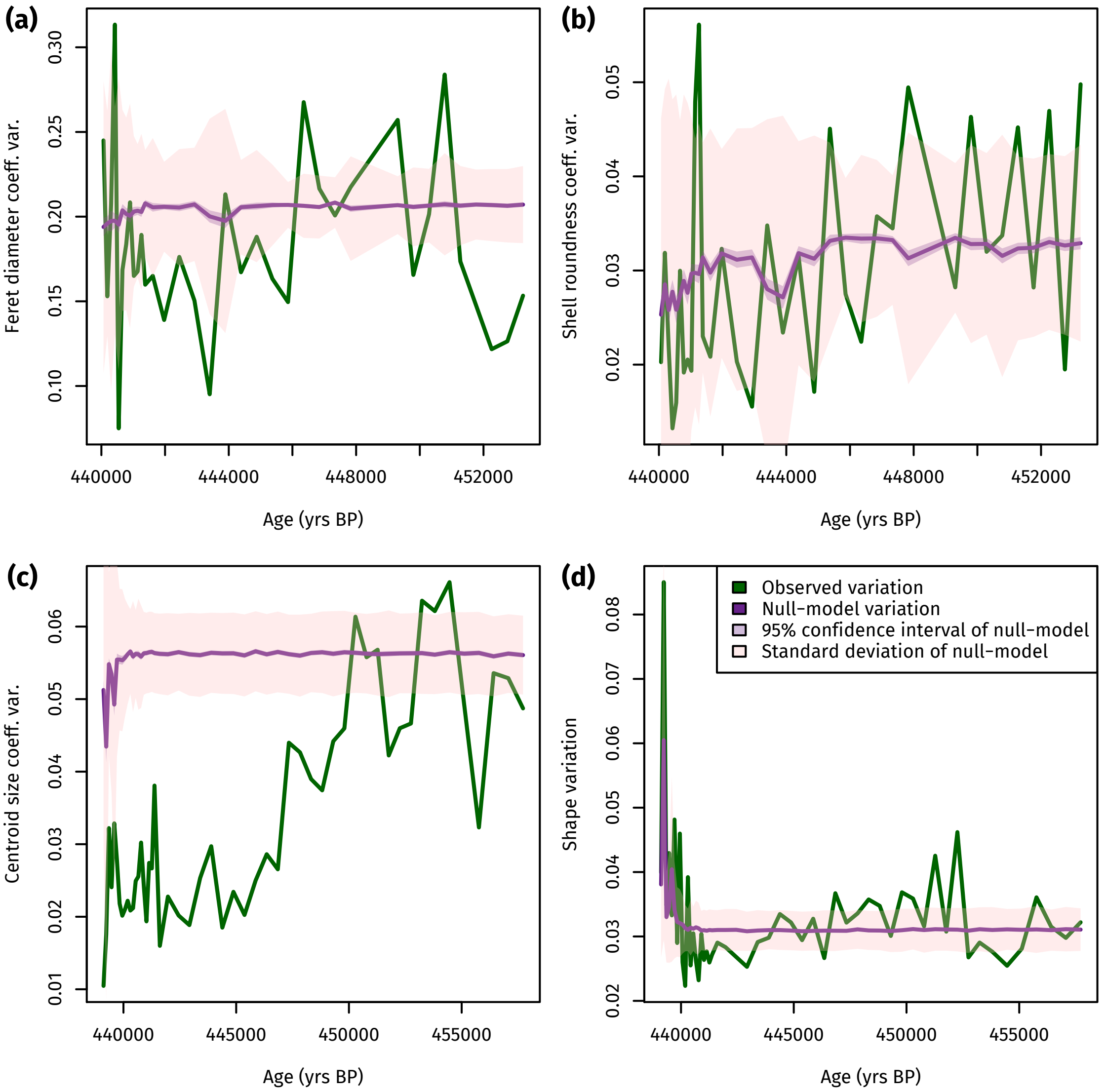

Figure S12. Results of a randomization approach to test trends in morphological variation during marine isotope stage 12 in the Red Sea. All variation parameters were calculated for randomly resampled samples (with replacement, 1000 replications), where the sample size was kept as in the original samples. (a–b) Shell size and roundness variation of *Orbulina universa* (large population only) as coefficient of variation. (c–d) Shell size (coefficient of variation) and shell shape variation (variance of the Riemannian shape distance from the grand mean) of *Trilobatus sacculifer*. In all cases, the random sampling (null-model) shows no trend at all, and the observed trend is always above the null-model earlier during the section and below the null-model when approaching the local extinction. This shows that the observed stabilization trend is stronger than can be expected by chance and is not biased by the decreasing sample sizes toward the local extinction.

3 Error discussion

Geometric morphometric analyses are error prone due to the large amount of manual steps involved, which can bias the reproducibility of the results. Amongst others, this includes the error of the measurement device and the measurer. We limited the device error by using the same microscope and camera for all photographs and using a constant magnification per species. The error by the measurer was limited by ensuring that all tasks were applied by the same researcher per specimen. Two other sources of error are only relevant for the geometric morphometric analyses of *Trilobatus sacculifer*. They were quantified using analysis of variances-based (ANOVA) approaches proposed by Yezerinac, Lougheed et al. [11], and are discussed in the following.

3.1 Error due to manual orientation of specimens

While all specimens per species were homogeneously oriented by the same researcher, eliminating a personal error term, a mis-orientation of specimens could still occur. To estimate that error, we repeatedly (eight times) reoriented three randomly selected specimens covering the whole observed size range, and estimated the landmark position error due to orientation. The analyses shows that manually orienting the specimens did not introduce a large error. The mean squares of the replicates (2219) is much smaller than the residual mean squares (23 739), with the ANOVA being highly insignificant ($p = 0.999$). This indicates a high reproducibility of landmarks regardless of small orientation errors, and the relative measurement error associated with specimen orientation sums up to only 1.07 %.

3.2 Error due to manual landmark placement

A second source of error is the problem of misplacing landmarks in the specimen images. Due to the large amount of samples it was not feasible to replicate landmark extraction for all samples, instead we replicated this step for two samples, one with on average very small specimens (1439.5–1440 cm) and one with on average very large specimens (1488–1488.5 cm). The reasoning behind this is that larger specimens have a higher effective resolution, because they contain more pixels under constant magnification, and morphological details are better visible in larger specimens when the magnification is kept constant. It is thus likely that the landmark extraction error is not independent of specimen size. For both samples we replicated the landmark extraction and calculated the session error (i.e. the average mismatch between replicates) and the individual error (i.e. the error per specimen) as well as the relative measurement error (Table S3). In both samples we observe that the residual mean squares (error variance) of the session is much larger than the session factor mean squares, and that the ANOVA results for the session error are insignificant, indicating a high replicability of landmark positions in different sessions. The individual error ANOVA is highly significant in both cases, with the individual factor mean squares (between-specimen variance) being much larger than the residual mean squares (within-specimen variance). This indicates that the observed differences between specimens are much larger than what could be explained by errors in landmark extraction. Accordingly, the relative measurement errors associated with landmark misplacement are very small, with 0.264 % for small specimens and 0.148 % for large specimens.

Table S3. Error calculation for landmark extraction in *Trilobtaus sacculifer* specimens from marine isotope stage 12 in the Red Sea, exemplarily performed on two samples with on average very small (1439.5–1440 cm) and very large (1488–1488.5 cm) specimens.

		1439.5–1440 cm	1488–1488.5 cm
Session error	Session factor mean squares	52	20
	Residual mean squares	4105	21194
	p-value	0.910	0.976
Individual error	Individual factor mean squares	8199	42357
	Residual mean squares	11	31
	p-value	< 0.001	< 0.001

3.3 Allometry analyses

One last potential problem in morphometric analyses, especially in geometric morphometrics, can be the influence of ontogeny on shape. This can be tested in two ways [12]. (1) One can adapt the univariate allometric equation and calculate the Riemannian shape distances between the smallest individual and all other individuals, and then calculate the regression between size and shape distance to the smallest individual. When doing this for our data for *T. sacculifer* (Fig. S13a) we find that the regression is insignificant ($p = 0.919$) and shell size explains less of the observed shape change than the null-model ($R^2 < -0.001$). (2) Alternatively, one can regress the partial warps of the landmark configuration on centroid size data in a multivariate regression approach to investigate the shape change as a whole dependent on size [12, eq. 11.4]. Doing so reveals a significant allometric component in *T. sacculifer* shape ($p < 0.001$), but it explains only 4.10 % of the observed shape changes and is thus practically negligible, because it cannot be responsible for the signals we see in the data. It is furthermore mainly limited to a widening of the aperture (Fig. S13b), which is no trend that we observe in the *T. sacculifer* population in association with increasing stress levels. We thus conclude that allometry is no problem in our analysis of *T. sacculifer* shape changes with environmental stress.

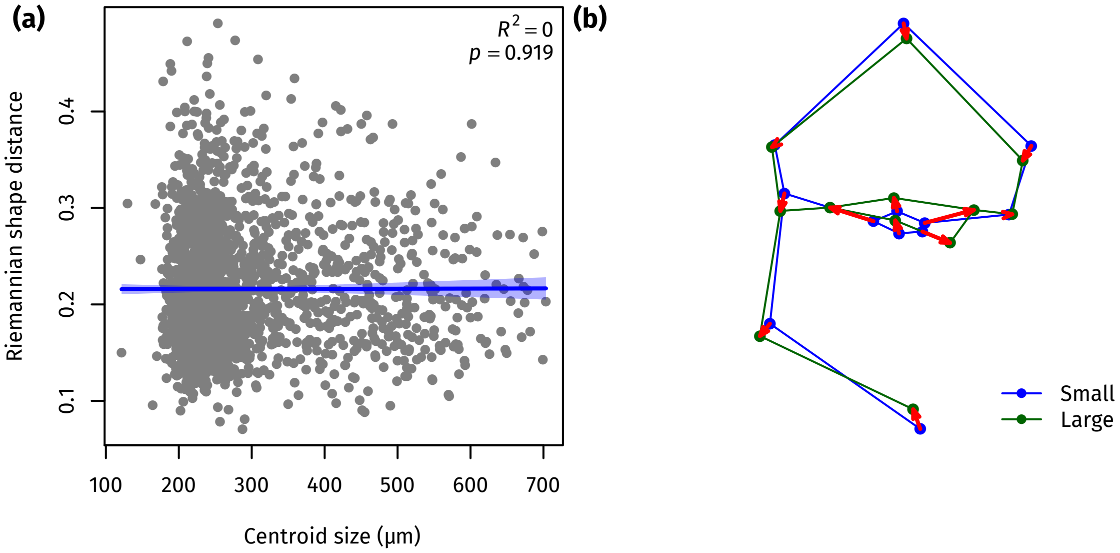

Figure S13. Allometry of *Trilobatus sacculifer* from marine isotope stage 12 in the Red Sea. (a) The univariate allometry approach shows no significant correlation between shape (expressed as Riemannian shape distance from the smallest individual) and size. (b) The shape change with ontogeny is nearly exclusively limited to a widening of the aperture.

References

1. Bookstein FL. Morphometric Tools for Landmark Data: Geometry and Biology. Cambridge, New York, Melbourne: Cambridge University Press, 1991. 435 p.
2. Reuss AE. Neue Foraminiferen aus den Schichten des österreichischen Tertiärbeckens. Denkschr Kaiserl Akad Wiss, Math–Naturwiss Classe. 1850; 1:365–390.
3. Banner FT, Blow WH. Some primary types of species belonging to the superfamily Globigerinaceae. Contrib Cushman Found Foraminiferal Res. 1960; 11(1):1–41.
4. Nelder JA, Wedderburn RWM. Generalized linear models. J R Stat Soc , Ser A: Gen. 1972; 135(3):370–384. http://www.jstor.org/stable/2344614.
5. Ellison AM. Effect of seed dimorphism on the density-dependent dynamics of experimental populations of *Atriplex triangularis* (chenopodiaceae). Am J Bot. 1987; 74(8):1280–1288. https://dx.doi.org/10.1002/j.1537-2197.1987.tb08741.x.
6. Hartigan JA, Hartigan PM. The dip test of unimodality. Ann Stat. 1985; 13(1):70–84. https://dx.doi.org/10.1214/aos/1176346577.
7. Kendall MG. A new measurement of rank correlation. Biometrika. 1938; 30(1–2):81–93. https://dx.doi.org/10.1093/biomet/30.1-2.81.
8. Theil H. A rank-invariant method of linear and polynomial regression analysis, iii. Proc K Ned Akad Wet. 1950; 53(9):1397–1412.
9. Sen PK. Estimates of the regression coefficient based on Kendall's tau. J Am Stat Assoc. 1968; 63(324):1379–1389. http://www.jstor.org/stable/2285891.

10. Anderson MJ. A new method for non-parametric multivariate analysis of variance. Austral Ecol. 2001; 26(1):32–46. https://dx.doi.org/10.1111/j.1442-9993.2001.01070.pp.x.

11. Yezerinac SM, Lougheed SC, Handford P. Measurement error and morphometric studies: Statistical power and observer experience. Syst Biol. 1992; 41(4):471–482. https://dx.doi.org/10.1093/sysbio/41.4.471.

12. Zelditch ML, Swiderski DL, Sheets HD. Geometric Morphometrics for Biologists: A Primer. 2nd ed. London, Waltham, San Diego: Academic Press, 2012. 478 p. http://booksite.elsevier.com/9780123869036/index.php.
